# Reduction of severe intraventricular hemorrhage, a tertiary single-center experience: incidence trends, associated risk factors, and hospital policy

**DOI:** 10.1007/s00381-020-04621-7

**Published:** 2020-05-04

**Authors:** Wafa Sattam M. Alotaibi, Nada S. Alsaif, Ibrahim A. Ahmed, Aly Farouk Mahmoud, Kamal Ali, Abdullah Hammad, Omar S. Aldibasi, Saif A. Alsaif

**Affiliations:** 1 King Saud bin Abdulaziz University for Health Sciences, Riyadh, Saudi Arabia; 2 Neonatal Intensive Care Department, King Abdulaziz Medical City, Riyadh, Saudi Arabia; 3 Medical Imaging Department, Pediatric Radiology, King Abdulaziz Medical City, Riyadh, Saudi Arabia; 4 Department of Bioinformatics and Biostatistics, King Abdullah International Medical Research Center, Riyadh, Saudi Arabia

**Keywords:** Neonatology, Prematurity, IVH, Neurodevelopmental

## Abstract

**Objectives:**

To determine the incidence, trends, maternal and neonatal risk factors of severe intraventricular hemorrhage (IVH) among infants born 24–32 weeks and/or < 1500 g, and to evaluate the impact of changing of hospital policies and unit clinical practice on the IVH incidence.

**Study design:**

Retrospective chart review of preterm infants with a gestational age (GA) of 24–32^6^ weeks and/or weight of < 1500 g born at King Abdulaziz Medical City–Riyadh (KAMC-R), Saudi Arabia, from 2016 to 2018. Multivariate logistic regression model was constructed to determine the probability of developing severe IVH and identify associations with maternal and neonatal risk factors.

**Results:**

Among 640 infants, the overall incidence of severe IVH was 6.4% (41 infants), and its rate decreased significantly, from 9.4% in 2016 to 4.5% and 5% in 2017 and 2018 (*p* = 0.044). Multivariate analysis revealed that caesarian section delivery decreased the risk of severe IVH in GA group 24–27 weeks (*p* = 0.045). Furthermore use of inotropes (*p* = 0.0004) and surfactant (*p* = 0.0003) increased the risk of severe IVH. Despite increasing use of inotropes (*p* = 0.024), surfactant therapy (*p* = 0.034), and need for delivery room intubation (*p* = 0.015), there was a significant reduction in the incidence of severe IVH following the change in unit clinical practice and hospital policy (*p* = 0.007).

**Conclusion:**

Cesarean section was associated with decreased all grades of IVH and severe IVH, while use of inotropes was associated with increased severe IVH. The changes in hospital and unit policy were correlated with decreased IVH during the study period.

**Electronic supplementary material:**

The online version of this article (10.1007/s00381-020-04621-7) contains supplementary material, which is available to authorized users.

## Introduction

Neurological sequelae are found in approximately 50–75% of preterm survivors with severe intraventricular hemorrhage (IVH); hence, it remains a significant public health concern worldwide [1].

According to the National Institute of Child Health and Human Development (NICHD) Neonatal Research Network, in 2016, 23.7% of sonograms of extremely low gestational age (GA) and VLBW infants indicated the development of IVH, where 31.6% of these cases were classified as severe IVH [2]. Recent data from the Korean Neonatal Network showed that the overall incidence of IVH in VLBW infants was 42.5%, while severe IVH was 10.3% [3], and few studies in the Middle East have reported that severe IVH occurs in 8.1% and as high as 11% among high-risk infants [4, 5].

Some studies examine the associated risk factors of IVH in preterm infants. It was found that antenatal steroid therapy use and birth via cesarean section were associated with decreased rates of severe IVH [6–12]. Other risk factors include hypotension and hypercapnia as they cause significant fluctuations in cerebral blood flow [7].

Different modalities have been used to decrease the rate of severe IVH, such as use of prophylactic indomethacin [13, 14], limiting the attempts of intubation for extremely preterm infants [15, 16] and minimizing the use of inotropes [17].

Jensen and Lorch [18, 19] concluded that very low birth weight neonates born between midnight and 07: 00 hrs are at increased risk for severe IVH and death or major neonatal morbidities. The American Academy of Pediatrics recommends 24-h in-house coverage by an attending physician in the intensive care unit (ICU) [20].

Thus, this study aims to determine the incidence, trends, and maternal and neonatal risk factors of severe intraventricular hemorrhage (IVH) among infants born 24–32 weeks [2] and/or < 1500 g and to evaluate the impact of changing hospital policies and unit practice on the IVH incidence.

## Method

This is a retrospective cohort review that was conducted in the neonatal intensive care unit (NICU) at King Abdulaziz Medical City (KAMC), Riyadh, Kingdom of Saudi Arabia. This NICU is a 76-bed level II and IIIC unit, with an average of 2300 admissions per annum.

Prior to 2017, neonatologists in our department used to do in-house service only during 08:00–17:00, and after-hours, the on-call neonatologist was to take calls from home. In January 2017, a new system of 24-h in-house neonatologist coverage was introduced.

The project and the study protocol were approved by the Institutional Review Board at KAMC-R.

The inclusion criteria were infants born between January 2016 and December 2018 at 24 to 32 weeks [2] GA and/or weighing less than or equal to 1500 g. Infants born outside of KAMC-R, those who died within 7 days of birth and those with major congenital anomalies were excluded from the study.

Data were retrieved from electronic medical records including maternal demographics such as antenatal steroid and magnesium sulfate use, mode of delivery, maternal hypertension or preeclampsia, and parity. Neonatal demographics including the infants’ GA based on the best estimate from an earlier obstetric scan, birth weight, gender, and the Apgar scores at 1 and 5 min (Table 1).

**Table 1:** Neonatal demographics

Cohort characteristics by IVH severity in 640 preterm infants (GA, 24–32 weeks)						
Variable	Count (%)/mean (SD)					
		No IVH	I and II	III and IV	Total	*p value*
Sex	Male	284 (79.11)	51 (14.21)	24 (6.69)	359	0.9433
	Female	223 (79.36)	41 (14.59)	17 (6.05)	281	
Gestational age (weeks)	24–25	28 (49.12)	14 (24.56)	15 (26.32)	57	*< .0001*
	26–27	44 (59.46)	17 (22.97)	13 (17.57)	74	
	28–29	92 (73.60)	25 (20)	8 (6.40)	125	
	30–32	343 (89.32)	36 (9.38)	5 (1.30)	384	
	Mean (SD)	29.89 (2.09)	28.52 (2.46)	26.65 (2.25)		
Birth weight (g)	500–750	36 (54.55)	16 (24.24)	14 (21.21)	66	*< .0001*
	751–1000	66 (68.04)	18 (18.56)	13 (13.40)	97	
	1001–1250	72 (70.59)	24 (23.53)	6 (5.88)	102	
	1251–1500	124 (85.52)	16 (11.03)	5 (3.45)	145	
	> 1500	209 (90.87)	18 (7.83)	3 (1.30)	230	
	Mean (SD)	1420.73	1185.58 (420.70)	986.82 (420.01)		
		− 430.35				

The italized values in Tables I and II with p <.05 indicate statistical significance

In addition to the demographics above, we also reviewed the need for delivery room intubation, need for surfactant therapy, HFOV/high FiO_2_ requirement > 0.8 in the first 24 h, use of inotropes within 72 h and hypercapnia (pCO_2_ > 65 mmHg) in two readings on arterial blood gas during the 1st week. Occurrence of thrombocytopenia (platelets < 100,000 per microliter of blood), severe metabolic acidosis (BE > 12 mmol/l) within first 7 days and culture positive sepsis during the course of hospital stay were also documented.

Initial cranial ultrasonographic scans were performed within 7 days of life according to the American Academy of Neurology [21]. The frequency of subsequent imaging is dictated by the severity of IVH detected on the first ultrasound scan, e.g., infants with grades III and IV IVH will have weekly ultrasound imaging as per our departmental policy. The severity of IVH is graded where grades I and II are categorized as mild IVH, and grades III and IV are severe IVH. We use Papille classification which states: “grade I IVH refers to bleeding confined to the germinal matrix; grade II indicates IVH occupied ≤50% of the lateral ventricle volume; grade III IVH occupied >50% of lateral ventricle volume with dilatation of ventricles; and grade IV IVH indicates the presence of an infarction and/or hemorrhage in the periventricular white matter” [22].

### Statistical analysis

Descriptive statistics were used to describe the demographic and clinical characteristics of the study sample. Categorical variables are reported as frequencies, and percentages and continuous variables are summarized as means with standard deviations. Chi-square and ANOVA tests were used to evaluate differences in the study variables among the study years (2016, 2017, and 2018) and according to IVH outcome status (no IVH, IVH I, or II and IVH III or IV).

A multivariate logistic regression model was applied to model the probability of devolving any grade of IVH (I, II, III, or IV) and the associations between devolving severe IVH (III or IV) and risk factors. All models were stratified by GA (24–27 weeks and 28–32 weeks). As our sample included mothers with multiple pregnancies, we divided the study variables according to maternal risk factors and neonatal risk factors.

For maternal risk factors, we modeled the probability of developing any grade of IVH and the probability of at least one infant developing severe IVH (*N* = 567 mothers). For the neonatal-associated risk factors, we modeled the probability of devolving any grade of IVH and the probability of developing severe IVH for each infant (*N* = 640 infants). All analyses were conducted using the Statistical Analysis System (SAS) software, version 9.4., SAS Institute, Inc., Cary, NC, USA. The level of significance was declared at *p* < .05.

## Results

**Fig. 1:**
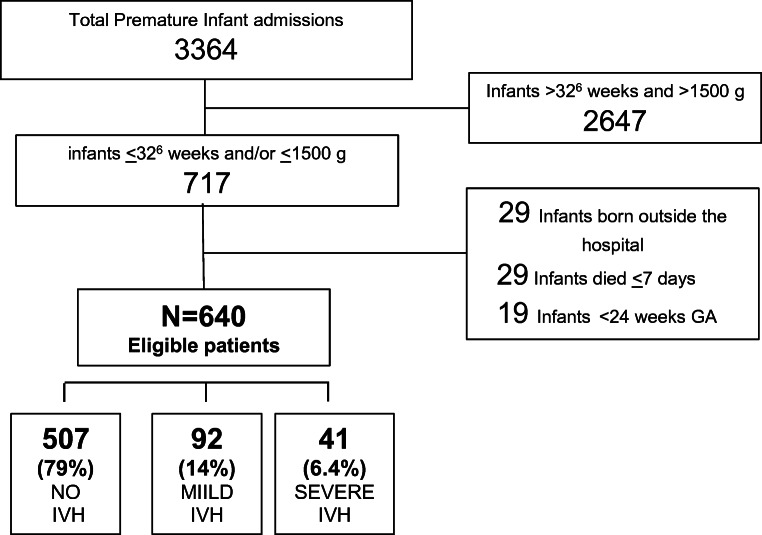
Flowchart of the study population

During the study period, 717 infants with GA of 24–32 [2] weeks and/or birth weight of < 1500 g were admitted to our NICU. Of those infants, 640 met the inclusion criteria for this study (Fig. 1). Four hundred seventy-seven mothers who gave birth to singletons and 90 had multiple pregnancies.

**Fig. 2:**
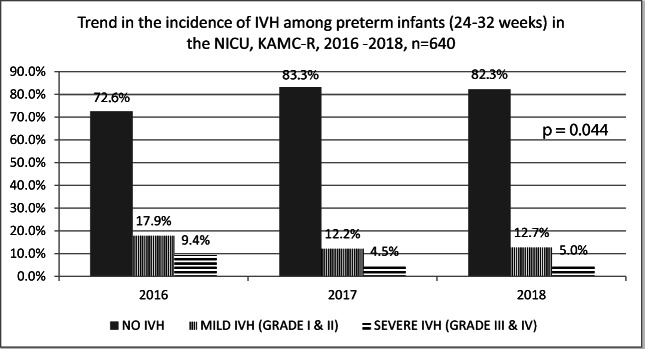
The trend in the incidence of IVH from 2016 to 2018

The overall incidence of IVH among the 640 infants was 21.4%, where mild IVH was 14%, while severe IVH was 6.4% (3.4% grade III and 2.9% grade IV IVH). Rates of IVH decreased significantly over time during the study period from 9.4% in 2016 to 4.5% in 2017 and 5% in 2018, with *p* = .044 (Fig. 2). Of the 6.4% infants who developed severe IVH, 90% survived to hospital discharge.

The rate of severe IVH was highest in the youngest GA subgroup (26.3%, 24–25 weeks), while it was significantly decreased in the older GA groups to 17.6% at 26–27 weeks, 6.4% at 28–29 weeks, and 1.3% at 30–32 weeks (*p* ≤ 0.0001).

The rate of severe IVH was 21% in infants with a birth weight of 500–750 g, 13% in the 751–1000 g, 6% in the 1001–1250 g, and 3% in the 1251–1500 g (*p* ≤ 0.0001).

There were no significant differences in antenatal steroids (*p* = 0.1121), antenatal magnesium sulfate use (*p* = 0.5619), and history of maternal hypertension (*p* = 0.2497) between the different grades of IVH. There was no statistically significant differences between singleton and multiple births with regard to IVH severity (*p* = 0.1306). Caesarian section delivery was associated with reduced incidence and severity of IVH (*p* < 0.001) (Table 2).

**Table 2:** Maternal and neonatal associated risk factors of IVH

	Count (%)/mean (SD)					
		No IVH	I and II	III and IV	Total	*p* value
Maternal risk factors						
Maternal antenatal steroid treatment	No	170 (75.89)	41 (18.30)	13 (5.80)	224	0.1121
	Yes	337 (81.01)	51 (12.26)	28 (6.73)	416	
Antenatal magnesium sulfate use	No	373 (79.03)	66 (13.98)	33 (6.99)	472	0.5619
	Yes	133 (79.64)	26 (15.57)	8 (4.79)	167	
Maternal hypertension/preeclampsia	No	455 (78.58)	84 (14.51)	40 (6.91)	579	0.2497
	Yes	52 (85.25)	8 (13.11)	1 (1.64)	61	
Mode of delivery	SVD	169 (69.83)	46 (19.01)	27 (11.16)	242	*< .0001*
	C/S	337 (84.92)	46 (11.56)	14 (3.52)	398	
Parity	Single	373 (78.20)	68 (14.26)	36 (7.55)	477	0.1306
	Multiple	134 (82.21)	24 (14.72)	5 (3.07)	163	
Neonatal risk factors						
Surfactant use	No	317 (85.68)	45 (12.16)	8 (2.16)	370	*< .0001*
	Yes	190 (70.37)	47 (17.41)	33 (12.22)	270	
Delivery room CPR	No	506 (79.43)	91 (14.29)	40 (6.28)	637	0.0835
	Yes	1 (33.33)	1 (33.33)	1 (33.33)	3	
Need for delivery room intubation	No	416 (84.38)	58 (11.76)	19 (3.85)	493	*< .0001*
	Yes	91 (61.90)	34 (23.13)	22 (14.97)	147	
High FiO_2_ (> 0.8) or need for HFOV in the first 24 h	No	482 (81.42)	77 (13.01)	33 (5.57)	592	*< .0001*
	Yes	25 (52.08)	15 (31.25)	8 (16.67)	48	
Use of inotropes within 72 h	No	461 (82.47)	75 (13.42)	23 (4.11)	559	*< .0001*
	Yes	46 (56.79)	17 (20.99)	18 (22.22)	81	
Hypercapnia (> 65) during the 1st week (2× blood gas)	No	454 (80.50)	75 (13.30)	35 (6.21)	564	*0.0409*
	Yes	48 (67.61)	17 (23.61)	6 (8.33)	72	
Metabolic acidosis (BE >− 12) during the 1st week	No	496 (79.87)	87 (14.01)	38 (6.12)	621	*0.0061*
	Yes	7 (46.67)	5 (33.33)	3 (20)	15	
Positive blood culture within 72 h of birth	No	503 (80.35)	85 (13.58)	38 (6.07)	626	*< .0001*
	Yes	4 (28.57)	7 (50)	3 (21.43)	14	
Platelets < 100,000 per microliter of blood (within 7 days of age)	No	431 (83.20)	66 (12.74)	21 (4.05)	518	*< .0001*
	Yes	76 (62.30)	26 (21.31)	20 (16.39)	122	
Apgar score (at 1 min)		6.05 (2.00)	5.23 (2.08)	4.37 (2.33)		*< .0001*
Apgar score (at 5 min)		7.81 (1.75)	7.22 (1.96)	6.29 (1.99)		*< .0001*

The italized values in Tables I and II with p <.05 indicate statistical significance

Need for delivery room intubation, surfactant therapy, HFOV or FiO_2_ requirement > 0.8 and inotropic support in the first 72 h were significantly associated with increased rates and severity of IVH (*P* < 0.001). In addition, thrombocytopenia (< 100,000/mcl), low Apgar scores at 1 and 5 min and positive blood culture during the course of hospital stay significantly increased the rates and severity of IVH (*p* < 0.001). Hypercapnia (*p* = 0.04) and severe metabolic acidosis (*p* = 0.006) within the first 7 days were also associated with increased risk and severity of IVH. Need for delivery room CPR (*p* = 0.08) was not associated with an increased risk nor severity of IVH (Table 2).

Multivariate stepwise analysis of the neonatal risk factors for IVH in all preterm infants stratified by GA groups showed that hypercapnia in two arterial blood gas readings during the 1st week (OR = 2.40, 95% CI 1.17–4.89, *p* = 0.01) and high FiO_2_ or the need for HFOV during the first 24 h (OR = 2.90, 95% CI 1.20–6.96, *p* = 0.01) was associated with increased risk of any grade of IVH in infants born at 28–32 weeks of gestation, while the use of inotropes was associated with increased IVH in infants born at 24–27 weeks’ gestation (OR = 2.60, 95% CI 1.20–5.62, *p* = 0.01) (Table 3).

**Table 3:** Multivariate analysis of neonatal and maternal risk factors associated with any grade of IVH among preterm Infants (24–32 weeks)

IVH (all)	24–27 weeks				28–32 weeks			
Variables	OR	95% CI (lower)	95% CI (upper)	*p* value	OR	95% CI (lower)	95% CI (upper)	*p* value
Hypercapnia (> 65) during the 1st week (2× blood gas)					2.40	1.17	4.89	0.01
Use of inotropes within 72 h	2.60	1.20	5.62	0.01				
High FiO_2_ (> 0.8) or the need for HFOV for the first 24 h					2.90	1.20	6.96	0.01
Apgar at 1 min					0.86	0.77	0.97	0.01
Maternal antenatal steroid treatment					0.55	0.32	0.95	0.03
Mode of delivery (C/S)	0.28	0.12	0.64	0.002				

Multivariate regression analysis of maternal-associated risk factors showed that delivery via caesarian section (OR = 0.28, 95% CI 0.12–0.64, *p* = 0.002) was associated with a decreased the risk of developing any grade of IVH among infants born at 24–27 weeks gestation. Maternal antenatal steroids was associated with decreased the risk of any grade of IVH in infants born at 28–32 weeks gestation (OR = 0.55, 95% CI 0.32–0.95, *p* = 0.03) (Table 3).

Multivariate regression analysis for maternal and neonatal risk factors for severe IVH showed that patients who received inotropes associated with an increased the risk of severe IVH among infants born at 24–27 weeks of gestation (OR = 3.97, 95% CI 1.62–9.73, *p* = 0.002) whereas the use of surfactant was associated with an increase of severe IVH in infants born at 28–32 weeks of gestation (OR = 6.60, 95% CI 1.79–24.33, *p* = 0.004).

A high 5-min (but not the first minute) Apgar score was also found to be associated with a decreased risk of severe IVH among relatively small patients born at 24–27 weeks’ gestation (OR = 0.80, 95% CI 0.66–0.97, *p* = 0.028).

Delivery via caesarian section (OR = 0.37, 95% CI 0.14–0.97, *p* = 0.04) was associated with a decreased severe IVH in infants born at 24–27 weeks of gestation (Table 4).

**Table 4:** Multivariate analysis of neonatal and maternal risk factors associated with severe IVH among preterm infants (24–32 weeks)

IVH (severe)	24–27 weeks				28–32 weeks			
Variables	OR	95% CI (lower)	95% CI (upper)	*p* value	OR	95% CI (lower)	95% CI (upper)	*p* value
Use of inotropes within 72 h	3.97	1.62	9.73	0.002				
Use of surfactant					6.60	1.79	24.33	0.004
Apgar at 5 min	0.80	0.66	0.97	0.028				
Mode of delivery (C/S)	0.37	0.14	0.97	0.04				

The implementation of full-time in-house neonatologist was associated with a significantly reduced incidence of all grades and severe IVH with *p* = .007 (Table 5).

**Table 5:** Incidence of severe IVH pre and post implementation of unit policy

IVH grades	2016	2017–2018	Total	*p* value
	No. (%)	No. (%)		
No IVH	162 (72.65)	345 (82.73)	507	0.007
I or II IVH	40 (17.94)	52 (12.47)	92	
Severe IVH	21 (9.42)	20 (4.80)	41	
Total	223	417	640	

## Discussion

The overall incidence of severe IVH in the study period was 6.4%. Of those infants, 3.4% had grade III IVH, and 2.9% had grade IV IVH. A study in Saudi Arabia reported the incidence of severe IVH to be 8.1% in infants less than 32 weeks gestation [4]. Most of our findings regarding neonatal-associated factors were consistent with those reported in the literature.

In our study, there were no gender differences in the incidence of severe IVH in preterm infants, which is consistent with the findings of Vogtmann [23] and Singh [24]. However, others [25, 26] have described higher incidence of severe IVH in preterm male infants.

We have found that antenatal steroid use decreased the likelihood the development of any grade of IVH in infants born at 28–32 weeks gestation. We did not find any association between antenatal steroid use and severe IVH in 24–27 weeks’ gestational age. Our antenatal steroid uptake is 65% of pregnant women during the study period. This low percentage might explain the lack of link between severe IVH and antenatal steroids in very premature infants described in other studies [8–11, 27].

We have also found that thrombocytopenia increased the risk of any IVH in all preterm infants < 33 weeks, and severe IVH only in the younger GA group. Lindern et al. [28] found that 44% of infants born at 32 weeks’ gestation had thrombocytopenia. Within that group, there was a 30% incidence of IVH versus 14% in those with normal platelet counts.

We have found that the need of inotropic support within 72 h of birth increased the risk of any and severe IVH in infants born between 24 and 27 weeks’ gestation. This is similar to what recently described by Abdulaziz et al.[17]. In their study, they found an independent association between the early use of inotropes and death and/or severe brain injury in preterm infants born at < 29 weeks’ gestation.

The use of inotropes could be a marker for the severity of a patient’s illness, but some studies have highlighted the independent role of vasopressors in compromising cerebral autoregulation [29, 30]. It was recently found that the use of inotropes, rather than hypotension itself, was associated with an increased risk of severe IVH after adjusting for confounding factors [16].

The Cochrane Database 2011 [31] stated that use of hydrocortisone to treat hypotension had an efficacy similar to that of dopamine. In fact, when compared to a placebo, hydrocortisone did not increase the risk of IVH or the rate of infant mortality.

One modality used to prevent severe IVH among low birth weight infants is the use of indomethacin prophylaxis; however, in our NICU, we do not use prophylactic indomethacin for IVH prevention. According to the Trial of Indomethacin Prophylaxis in Preterms (TIPP) study, prophylactic indomethacin reduced the risk of severe IVH from 13 to 9% ((aOR) 0.6, 95% CI 0.4–0.9) but did not reduce the risk of the composite outcome of death or neurodevelopmental impairment (NDI) [13].

Some investigators have suggested that the selective use of prophylactic indomethacin in infants at high risk for severe IVH may have a greater treatment effect for preventing severe IVH and reducing the risk of the composite outcome of death or NDI[24, 25, 32]. However, a post hoc analysis of the TIPP study performed by Elizabeth et al. [14] did not support the selective use of prophylactic indomethacin treatment to improve long-term outcomes in VLBW infants at high risk of severe IVH.

In our study, delivery via caesarian section decreases the risk of any and severe IVH, among the young GA group (24–27 weeks). This was in line with other studies that found vaginal delivery increased the risk of IVH in infants born at less than 1500 g [12, 33].

Dani et al. [12] performed a multivariate analysis and reported that caesarian deliveries (RR 0.42, 95% CI 0.28–0.63) independently decreased the risk of developing IVH, compared with vaginal deliveries which was associated with an increased rate of each grade of IVH, but the increase was statistically significant was for grade III IVH (18% vs. 2%, *p* < 0.0001) and all grades of IVH together (45% vs. 20%, *p* < 0.0001) [12].

However, the Canadian Paediatric Society released a statement in 2019 that there is insufficient evidence to recommend routine caesarian section for women in preterm labor, unless the fetus is malpresenting [34].

Recently, a survey was conducted to identify variations in unit-level healthcare professionals’ availability for preterm neonates < 29 weeks gestation among 10 International Network for Evaluation of Outcomes (iNeo). Overall, they found that 55% of units had an in-house neonatologist 24 h a day. This ranged from 30% of units in Australia-New Zealand and Canada to 100% in Tuscany, Italy [20].

Lee et al. [35] reported that neonates born at < 32 weeks’ gestation and admitted at night had a 60% higher mortality than neonates admitted during the day, and an in-house neonatal fellow or attending neonatologist at night may reduce the odds of mortality by half.

A recent report by Lodha et al. [36] concluded in-house neonatologist coverage was associated with a reduction in the duration of mechanical ventilation in extremely preterm neonates. However, there was no difference in long-term outcomes.

In relation to these reports, the decrease in severe IVH in our center may be explained by the implementation of in-house neonatologist coverage*,* whereby consultants are physically available 24 h a day, 7 days a week.

This ensures the best quality of care is provided, during or after the golden hour. For instance, the consultants’ presence guarantees compliance with our safety protocols, which include delayed cord clamping before delivery and intubating infants weighing less than 1000 g done by senior, experienced physicians.

In early 2017, we implemented a policy that allows junior physicians (pediatric residents) to attempt intubation in NICU or labor and delivery room only infants with relatively high birth weight (more than 1500 g) and a relatively old GA (more than 32 weeks) and for those who received premedication.

Recently, it was found that increased intubation attempts have been associated with an increased incidence of severe IVH in infants with a birth weight less than 1500 g [37]. Intubation after birth has been found to be associated with IVH, with an OR (adjusted for low GAs) of 7.50 (95% CI 4.56–12.35) [15]. More recent study found that in infants with a birth weight < 1500 g, the number of intubation attempts in the delivery room was significantly increased in those with severe IVH (OR 1.31, 95% CI 1.05–1.64, *p* = 0.016) [16].

This study has some limitations. Firstly, it was a retrospective cohort review, with only 1 year (2016) before the implementation of new clinical practices. Antenatal steroid uptake of pregnant women was low during the study period, which might explain the lack of link between severe IVH and antenatal steroids in very premature infants. Furthermore, there was a lack of information on either complete, incomplete, or repeated antenatal steroid courses. Infants born outside of KAMC-Riyadh were also excluded from this study, as infants are transferred to our institution when they are older than 2 weeks of age, and therefore their conditions could not be reflecting our practice. Nineteen infants born at less than 24 weeks were excluded from this study as our guidelines on resuscitation of premature infants support those who are born at 24 weeks gestation or more.

In our study, we examined different contributing factors, especially antenatal steroid use and caesarian section delivery, and we found no significant difference among the study years. The same was also true for some major neonatal contributing factors, such as GA, birth weight, low platelet count, positive blood culture results in the first 72 h, hypercapnia, and the need for high FiO_2_ or HFOV.

However, despite the increased rates of delivery room intubation, inotrope, and surfactant use over the years in our institution, the incidence of severe IVH declined from 9.4% in 2016 to 4.8% in 2017–2018 (*p* = 0.007).

In conclusion, the trend of severe IVH significant drop could be due to various changes in clinical practices in our institution, mainly 24 h in-house neonatologist coverage and the limiting intubation attempts in preterm infants. However, a comparative study of at least 3 years before and after changes in policies and practices in our unit needed to determine its impact on severe IVH and other neonatal outcomes.

## Electronic supplementary material

ESM 1 (DOCX 54 kb)

ESM 2 (XLSX 121 kb)

## Abbreviations

ANOVA: Analysis of variance
C/S: Caesarian section
CPR: Cardiopulmonary resuscitation
GA: Gestational age
HFOV: High-frequency oscillatory ventilation
IVH: intraventricular hemorrhage
KAMC: King Abdulaziz Medical City
NICHD: National Institute of Child Health and Human Development
NICU: Neonatal intensive care unit
TIPP: Trial of Indomethacin Prophylaxis in Preterm
VLBW: Very low birthweight
