# Supplementary material for: Reduction of severe intraventricular hemorrhage, a tertiary single-center experience: incidence trends, associated risk factors, and hospital policy
Source: Childs Nerv Syst. 2020 May 4;36(12):2971–9. doi: 10.1007/s00381-020-04621-7 (PMC7649152; doi:10.1007/s00381-020-04621-7)
Supplement: Supplementary file 1 — (DOCX 54 kb) [file 381_2020_4621_MOESM1_ESM.docx]

**Supplementary Materials:**

**Table I. Demographic and clinical characteristics of the study subjects**

| ***Frequencies of each variable (N = 640)*** | | | |
| --- | --- | --- | --- |
| **Variable** |  | **Count/Mean** | **(%)/SD** |
| IVH (by severity) | No | 507 | 79.22 |
|  | Grade I or II | 92 | 14.38 |
|  | Severe | 41 | 6.41 |
| IVH (All) | No | 507 | 79.22 |
|  | Yes | 133 | 20.78 |
| Sex | Male | 359 | 56.09 |
|  | Female | 281 | 43.91 |
| Gestational age, weeks | 24-25 | 57 | 8.91 |
|  | 26-27 | 74 | 11.56 |
|  | 28-29 | 125 | 19.53 |
|  | 30-32 | 384 | 60 |
| Birth weight, g | 500-750 | 66 | 10.31 |
|  | 751-1000 | 97 | 15.16 |
|  | 1001-1250 | 102 | 15.94 |
|  | 1251-1500 | 145 | 22.66 |
|  | > 1500 | 230 | 35.94 |
| Maternal antenatal steroid treatment | No | 224 | 35 |
|  | Yes | 416 | 65 |
| Antenatal magnesium sulfate | No | 472 | 73.87 |
|  | Yes | 167 | 26.13 |
| Maternal hypertension/preeclampsia | No | 579 | 90.47 |
|  | Yes | 61 | 9.53 |
| Mode of delivery | SVD | 242 | 37.81 |
|  | C/S | 398 | 62.19 |
| Use of surfactant | No | 370 | 57.81 |
|  | Yes | 270 | 42.19 |
| Delivery room CPR | No | 637 | 99.53 |
|  | Yes | 3 | 0.47 |
| Need for delivery room intubation | No | 493 | 77.03 |
|  | Yes | 147 | 22.97 |
| Parity | Single | 477 | 74.53 |
|  | Multiple | 163 | 25.47 |
| High FiO_2_ (>0.8) or need for HFOV for the first 24 hours | No | 592 | 92.5 |
|  | Yes | 48 | 7.5 |
| Use of inotropes within 72 hours | No | 559 | 87.34 |
|  | Yes | 81 | 12.66 |
| Hypercapnia (>65) during the 1st week (2x blood gas) | No | 564 | 88.68 |
|  | Yes | 72 | 11.32 |
| Metabolic acidosis (BE > -12) during the 1^st^ week | No | 621 | 97.64 |
|  | Yes | 15 | 2.36 |
| Positive blood culture within 72 hours of birth | No | 626 | 97.81 |
|  | Yes | 14 | 2.19 |
| Platelets <100,000 per microliter of blood (within 7 days of age) | No | 518 | 80.94 |
|  | Yes | 122 | 19.06 |
| Apgar score (at 1 min) |  | 5.82 | 2.09 |
| Apgar score (at 5 min) |  | 7.62 | 1.84 |
| Birth year | 2016 | 223 | 34.84 |
|  | 2017 | 197 | 30.78 |
|  | 2018 | 220 | 34.38 |
|  | Total | 640 |  |

**Table II. Demographic and clinical characteristics of the study subjects stratified by year.**

| *Frequencies of each variable by year (N = 640)* | | | | | | | |
| --- | --- | --- | --- | --- | --- | --- | --- |
| Variable |  | | Count(%)/Mean(SD) | | |  |  |
|  | Year | 2016 | | 2017 | 2018 | Total | *p-value* |
| IVH (severity) | No | 162(72.65) | | 164(83.25) | 181(82.27) | 507 | ***0.044*** |
|  | Grade I or II | 40(17.94) | | 24(12.18) | 28(12.73) | 92 |  |
|  | Severe | 21(9.42) | | 9(4.57) | 11(5.00) | 41 |  |
|  | Total | 223 | | 197 | 220 | 640 |  |
| IVH (all) | No | 162(72.65) | | 164(83.25) | 181(82.27) | 507 | ***0.0109*** |
|  | Yes | 61(27.35) | | 33(16.75) | 39(17.73) | 133 |  |
|  | Total | 223 | | 197 | 220 | 640 |  |
| Sex | Male | 120(53.81) | | 101(51.27) | 138(62.73) | 359 | ***0.0436*** |
|  | Female | 103(46.19) | | 96(48.73) | 82(37.27) | 281 |  |
|  | Total | 223 | | 197 | 220 | 640 |  |
| Gestational age | 24-25 | 18(8.07) | | 20(10.15) | 19(8.64) | 57 | 0.9227 |
|  | 26-27 | 29(13.00) | | 23(11.68) | 22(10.00) | 74 |  |
|  | 28-29 | 45(20.18) | | 39(19.80) | 41(18.64) | 125 |  |
|  | 30-32 | 131(58.74) | | 115(58.38) | 138(62.73) | 384 |  |
|  | Total | 223 | | 197 | 220 | 640 |  |
| Birth weight, g | 500-750 | 21(9.42) | | 21(10.66) | 24(10.91) | 66 | 0.2423 |
|  | 751-1000 | 40(17.94) | | 28(14.21) | 29(13.18) | 97 |  |
|  | 1001-1250 | 35(15.70) | | 31(15.74) | 36(16.36) | 102 |  |
|  | 1251-1500 | 40(17.94) | | 42(21.32) | 63(28.64) | 145 |  |
|  | > 1500 | 87(39.01) | | 75(38.07) | 68(30.91) | 230 |  |
|  | Total | 223 | | 197 | 220 | 640 |  |
| Maternal antenatal steroid treatment | No | 90(40.36) | | 68(34.52) | 66(30.00) | 224 | 0.0723 |
|  | Yes | 133(59.64) | | 129(65.48) | 154(70.00) | 416 |  |
|  | Total | 223 | | 197 | 220 | 640 |  |
| Antenatal magnesium sulfate | No | 181(81.53) | | 131(66.50) | 160(72.73) | 472 | ***0.002*** |
|  | Yes | 41(18.47) | | 66(33.50) | 60(27.27) | 167 |  |
|  | Total | 222 | | 197 | 220 | 639 |  |
| Maternal hypertension/preeclampsia | No | 209(93.72) | | 174(88.32) | 196(89.09) | 579 | 0.1182 |
|  | Yes | 14(6.28) | | 23(11.68) | 24(10.91) | 61 |  |
|  | Total | 223 | | 197 | 220 | 640 |  |
| Mode of delivery | SVD | 81(36.32) | | 72(36.55) | 89(40.45) | 242 | 0.6073 |
|  | C/S | 142(63.68) | | 125(63.45) | 131(59.55) | 398 |  |
|  | Total | 223 | | 197 | 220 | 640 |  |
| Use of surfactant | No | 141(63.23) | | 100(50.76) | 129(58.64) | 370 | ***0.0341*** |
|  | Yes | 82(36.77) | | 97(49.24) | 91(41.36) | 270 |  |
|  | Total | 223 | | 197 | 220 | 640 |  |
| Delivery room CPR | No | 223(100.00) | | 195(98.98) | 219(99.55) | 637 | 0.3147 |
|  | Yes | 0(0) | | 2(1.02) | 1(0.45) | 3 |  |
|  | Total | 223 | | 197 | 220 | 640 |  |
| Need for delivery room intubation | No | 186(83.41) | | 142(72.08) | 165(75.00) | 493 | ***0.0152*** |
|  | Yes | 37(16.59) | | 55(27.92) | 55(25.00) | 147 |  |
|  | Total | 223 | | 197 | 220 | 640 |  |
| Parity | Single | 158(70.85) | | 138(70.05) | 181(82.27) | 477 | ***0.0049*** |
|  | Multiple | 65(29.15) | | 59(29.95) | 39(17.73) | 163 |  |
|  | Total | 223 | | 197 | 220 | 640 |  |
| High FiO_2_ (>0.8) or need for HFOV for the first 24 hours | No | 205(91.93) | | 184(93.40) | 203(92.27) | 592 | 0.8386 |
|  | Yes | 18(8.07) | | 13(6.60) | 17(7.73) | 48 |  |
|  | Total | 223 | | 197 | 220 | 640 |  |
| Use of inotropes within 72 hours | No | 205(91.93) | | 164(83.25) | 190(86.36) | 559 | ***0.0245*** |
|  | Yes | 18(8.07) | | 33(16.75) | 30(13.64) | 81 |  |
|  | Total | 223 | | 196 | 220 | 640 |  |
| Hypercapnia (>65) during the 1st week (2x blood gas) | No | 197(89.95) | | 169(85.79) | 198(90.00) | 564 | 0.3044 |
|  | Yes | 22(1.83) | | 28(14.21) | 22(10.00) | 72 |  |
|  | Total | 219 | | 195 | 219 | 636 |  |
| Metabolic acidosis (BE > -12) during the 1^st^ week | No | 215(98.17) | | 188(95.43) | 218(99.09) | 621 | ***0.0397*** |
|  | Yes | 4(26.67) | | 9(4.57) | 2(0.91) | 15 |  |
|  | Total | 219 | | 196 | 220 | 635 |  |
| Positive blood culture within 72 hours of birth | No | 219(98.21) | | 193(97.97) | 214(97.27) | 626 | 0.7851 |
|  | Yes | 4(1.79) | | 4(2.03) | 6(2.73) | 14 |  |
|  | Total | 223 | | 197 | 220 | 640 |  |
| Platelets <100,000 per microliter of blood (within 7 days of age) | No | 178(79.82) | | 166(84.26) | 174(79.09) | 518 | 0.3536 |
|  | Yes | 45(20.18) | | 31(15.74) | 46(20.91) | 122 |  |
|  | Total | 223 | | 197 | 220 | 640 |  |
| Apgar score (at 1 min) |  | 5.84(1.95) | | 5.70(2.33) | 5.92(2.00) |  | 0.5399 |
| Apgar score (at 5 min) |  | 7.65(1.50) | | 7.45(2.21) | 7.76(1.7) |  | 0.2108 |
|  | Total |  | |  |  |  |  |

**Figure I. Incidence of IVH in very low birth weight babies in the NICU, KAMC-R**

**Figure II. Incidence of IVH according to birth weight subgroup**

**Figure III. Incidence of IVH according to GA subgroup**
